# Experience With the Wearable Cardioverter-Defibrillator in Patients at High Risk for Sudden Cardiac Death

**DOI:** 10.1161/CIRCULATIONAHA.115.019124

**Published:** 2016-08-29

**Authors:** Nadine K. Wäßnig, Michael Günther, Silvio Quick, Christian Pfluecke, Fabian Rottstädt, Steven J. Szymkiewicz, Steven Ringquist, Ruth H. Strasser, Uwe Speiser

**Affiliations:** From Technische Universität Dresden, Heart Center Dresden, University Hospital, Department of Internal Medicine and Cardiology, Dresden, Germany (N.W., M.G., S.Q., C.P., F.R., R.S., U.S.); and ZOLL, Pittsburgh, PA (S.S., S.R.).

**Keywords:** arrhythmias, cardiac, death, sudden, cardiac, electric countershock, heart failure, myocarditis

## Abstract

**Background:**

This study evaluated the wearable cardioverter-defibrillator (WCD) for use and effectiveness in preventing sudden death caused by ventricular tachyarrhythmia or fibrillation.

**Methods:**

From April 2010 through October 2013, 6043 German WCD patients (median age, 57 years; male, 78.5%) were recruited from 404 German centers. Deidentified German patient data were used for a retrospective, nonrandomized analysis.

**Results:**

Ninety-four patients (1.6%) were treated by the WCD in response to ventricular tachyarrhythmia/fibrillation. The incidence rate was 8.4 (95% confidence interval, 6.8–10.2) per 100 patient-years. Patients with implantable cardioverter-defibrillator explantation had an incidence rate of 19.3 (95% confidence interval, 12.2–29.0) per 100 patient-years. In contrast, an incidence rate of 8.2 (95% confidence interval, 6.4–10.3) was observed in the remaining cardiac diagnosis groups, including dilated cardiomyopathy, myocarditis, and ischemic and nonischemic cardiomyopathies. Among 120 shocked patients, 112 (93%) survived 24 hours after treatment, whereas asystole was observed in 2 patients (0.03%) with 1 resulting death.

**ConclusionS:**

This large cohort represents the first nationwide evaluation of WCD use in patients outside the US healthcare system and confirms the overall value of the WCD in German treatment pathways.

## Abstract 2

Supplemental Digital Content is available in the text.

**Editorial, see p 644**

Previous studies have established the safety and efficacy of the wearable cardioverter-defibrillator (WCD) as a noninvasive alternative for patients who are suspected of being at temporary risk for sudden cardiac death (SCD) caused by ventricular fibrillation (VF) or ventricular tachyarrhythmia (VT).^1,2^ As demonstrated by large landmark trials, patients with ischemic cardiomyopathy (ICM) or nonischemic cardiomyopathy (NICM) and left ventricular ejection fraction (LVEF) ≤35% are at high risk for SCD and benefit from implantable cardioverter-defibrillator (ICD) therapy.^3–5^ In ≥40% of patients, however, LVEF may recover.^6,7^ As a result, ICD implantation is not indicated during the first 40 days after acute myocardial infarction (MI) or <3 months after coronary artery bypass grafting.^8,9^ In contrast, the WCD has been used successfully in patients during the first 40 days after acute MI and during the first 90 days after revascularization.^10^ Additional WCD uses fall into other transitional SCD risk categories such as postponed ICD implantation or reimplantation, early after the initial diagnosis of NICM, myocarditis, and during optimization of heart failure medical treatment.^11^ Capable of VT/VF detection and termination, the fully automated WCD protects outpatients and enables survival in patients experiencing otherwise fatal arrhythmic events independently of patient or bystander intervention.^11,12^

Despite extensive use of the WCD, evidence of its effectiveness when prescribed to patients outside the US healthcare system is lacking. To address this concern, the present nonrandomized, observational study investigated WCD use in a large cohort, including the types of cardiac patients currently receiving a WCD prescription, heart rhythm associated with delivery of electric shocks, conversion success, and survival outcome 24 hours after treatment.

## Methods

### Informed Consent

All patients signed consent to use their data for research purposes and quality monitoring when they received the WCD. Deidentified German patient data were used for a retrospective, nonrandomized analysis.

### Cohort

From April 2010 through October 2013, 6043 German WCD patients from all 16 German states were accrued. All patients were registered into the LifeVest Network, a registry maintained by the manufacturer of the WCD (ZOLL, Pittsburgh, PA). The German national database recorded demographics (eg, sex and age), cardiovascular indication leading to WCD prescription, defibrillation treatments, and daily wear times. Patient demographic data and the cardiac indication for WCD prescription were abstracted from physician medical orders.

#### Cardiac Indications

Patients were grouped into 8 cardiovascular indications reflecting diagnoses recorded for reimbursement billing purposes in Germany (Table 1), disease origin, and onset of symptoms leading to WCD prescription and wear. Patients were assigned to a single cardiovascular category with the use of the hierarchy and definitions below.

**Table 1.**
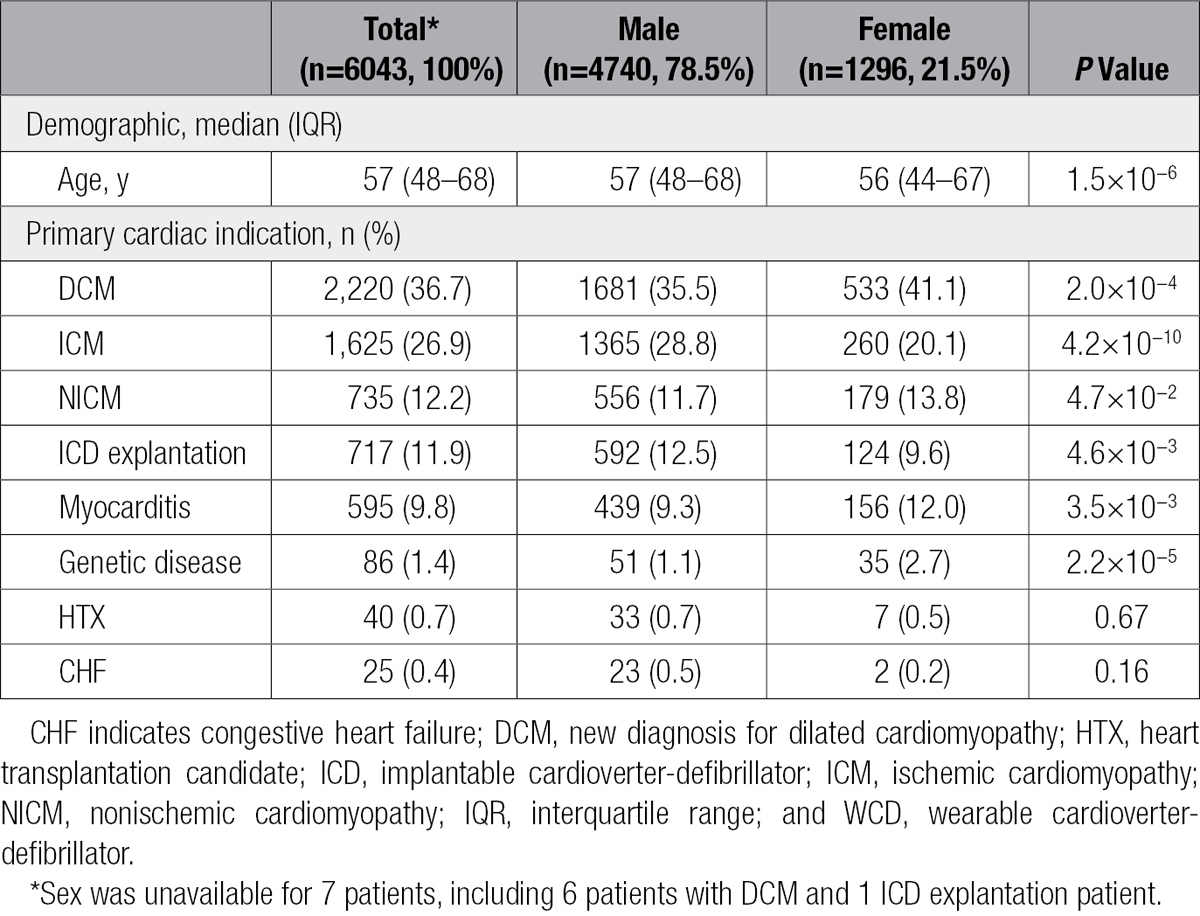
Baseline Patient Characteristics and Indication Leading to WCD Prescription

- ICD explantation: ICD explantation patients were those in whom a previously implanted ICD was removed but in whom reimplantation was delayed. The diagnosis leading to the original ICD implantation and the reason for reimplantation delay were unavailable for analysis.
- Congestive heart failure (CHF): Patients with CHF were under long-term heart failure medical therapy (>9 months) with a history of heart failure and otherwise met indications for an ICD but were not implanted because of comorbid conditions.
- Heart transplantation: Heart transplantation patients were on the transplant waiting list. The underlying cardiac condition necessitating the transplantation was unavailable for analysis.
- ICM: Patients with ICM had coronary artery disease and low LVEF. This group included both patients within 40 days after MI and patients within 90 days after revascularization.
- Dilated cardiomyopathy (DCM): Patients with DCM had newly diagnosed heart failure (within 90 days of the start of heart failure therapy) and had characteristic chamber enlargement.
- NICM: Patients with NICM did not meet the characteristics of ICM or DCM and were <90 days from diagnosis.
- Myocarditis: Patients with myocarditis had documented inflammation of the heart muscle, including viral infections, bacterial infections, and giant-cell myocarditis.
- Genetic disease: Patients with genetic disease had evidence of familial SCD syndromes such as long- or short-QT syndrome or Brugada syndrome.

### Adherence

Patient use data were obtained from the LifeVest Network database. Time of wear for individual days was recorded as seconds. Days of wear was defined as the sum of days in which the WCD was worn for >15 minutes. WCD daily use is reported as the ratio of the sum of hours and the sum of days minus 1. The 1-day adjustment is a correction for partial days available on the first and last days of prescribed wear.

### ECG Records

ECG data from WCD-detected events were obtained from the LifeVest Network database as PDF files. Analysis was performed by physicians of the University Dresden Heart Center. The ECGs of all patients, both treated and nontreated recordings, were reviewed and analyzed.

### Definitions

Appropriate treatments were defined as biphasic shocks delivered in response to an episode of VT with monomorphic or polymorphic characteristics or VF. Inappropriate treatments were WCD shocks delivered because of signal artifact or supraventricular tachycardia.

Asystole episodes were defined as periods of no discernable cardiac electric activity.

Interpretation of patient treatment data, including the review of ECG records, was performed by the authors.

### Post-Treatment Follow-Up

Technical support representatives used by ZOLL investigated all WCD treatments and spoke directly with patients who received a WCD shock or with the physicians caring for those patients. Outcomes were categorized, with survival defined as alive 24 hours after receiving a WCD shock.

### Statistical Analysis

Statistical analysis was performed by clinical statisticians from the University of Dresden who used the R statistical computing language.^13^ Analysis of categorical data was performed with the χ^2^ test. The frequency of cases is reported as 100 times the number of appropriately treated patients per total patients wearing the WCD. The mean incidence rate, in units of appropriately treated patients per 100 patient-years of WCD wear, was determined with the R package epicalc.^14^

## Results

### Patient Characteristics

From April 2010 through October 2013, a total of 6043 patients were prescribed and wore the WCD in Germany (Table 1). Complete case data consisting of age, sex, and cardiac diagnosis leading to WCD prescription were available for 6033 patients (99.8%). Patients were accrued from 404 German centers, with no single location contributing >4% of the patient population. Patients were recruited from each of the 16 German states, with the largest groups of patients residing in the most populous states such as North Rhine-Westphalia (1086, 18.0%) and Bavaria (742, 12.3%). Saarland had the fewest number of patients (26, 0.4%).

Patients were grouped into 8 cardiovascular indications (Table 1). The largest patient group was made up of those with newly diagnosed DCM (37%). ICM accounted for 27% of patients, including 43% who experienced MI within 40 days of WCD prescription, 44% who had received a percutaneous coronary intervention within 3 months, and 13% who had a coronary artery bypass graft within 3 months. In total, 12% of patients wore the WCD after ICD explantation. Patients with NICM accounted for an additional 12%. In 10% of patients, myocarditis was the reason for use. The remaining 2.5% of WCDs were prescribed for genetic disease (1.4%), heart transplantation listing (0.7%), or CHF (0.4%).

The German WCD population consisted of 78.5% male and 21.5% female patients with a median age of 57 years (interquartile range, 48–68 years; Table 1). The frequency distribution for reasons leading to WCD prescription differed between men and women, with the greatest difference occurring within ICM (28.8% of male prescriptions and 20.1% of female prescription). In contrast, female WCD users were more likely to be DCM and myocarditis patients.

### Patient WCD Use Data

As summarized in Table 2, the median for the German national cohort was 23.1 hours (interquartile range, 21.0–23.7 hours) of daily use and 59 total days (interquartile range, 33–90 total days) of wear. Patients had similar hours of daily use regardless of sex, age, or reason for WCD prescription.

**Table 2.**
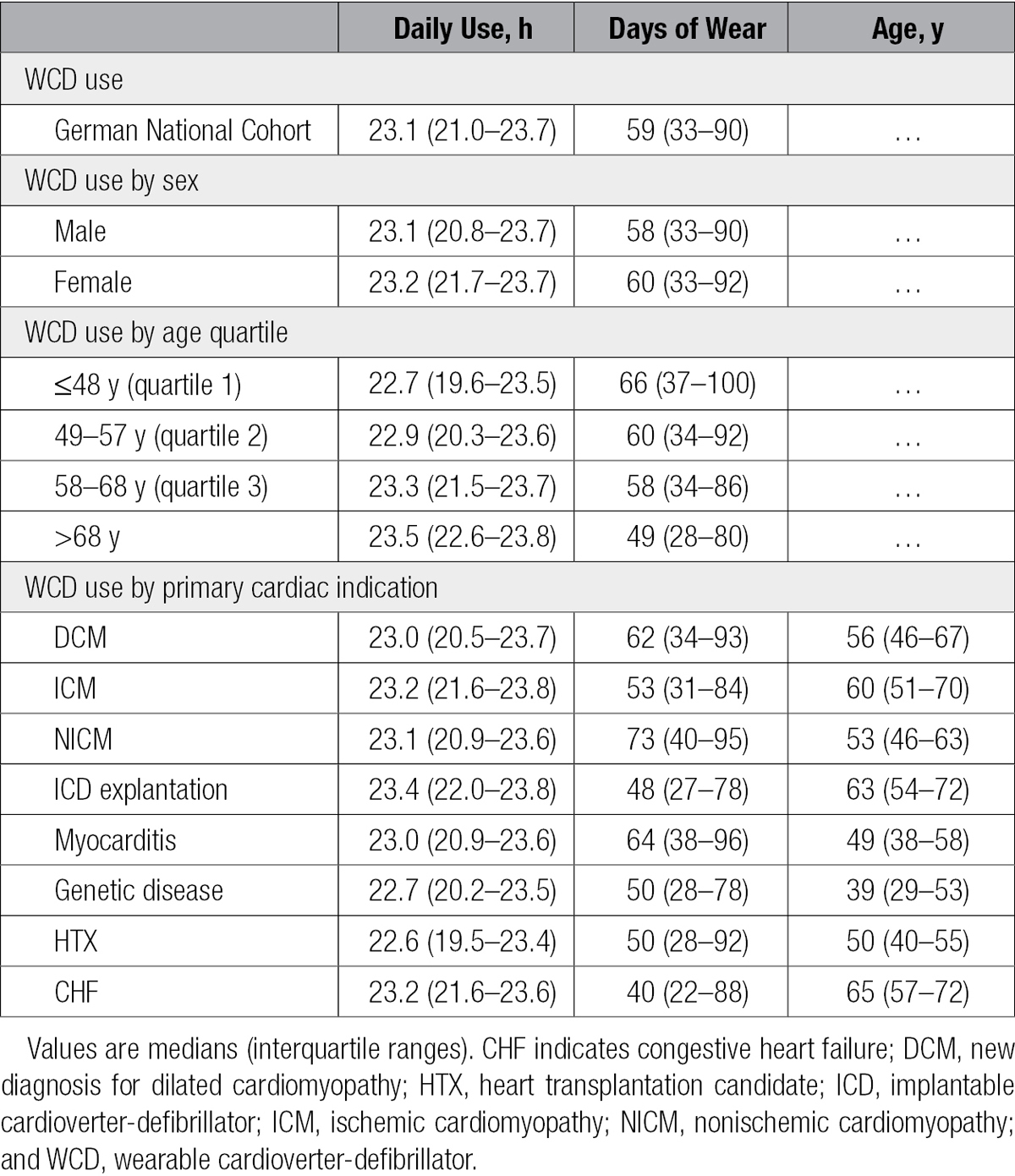
WCD Use by Sex, Age, and Reason for Prescription

In contrast, total days of prescribed WCD wear, although similar between the sexes, varied among patients by age (Table 2). For instance, when patients were grouped by age quartile, those patients ≤48 years of age (first age quartile) wore the WCD for a median of 66 days, whereas patients >68 years of age (fourth quartile) wore the WCD for a median of 49 days, a difference of 17 days of wear.

Likewise, differences in total days of prescribed wear were observed in patients grouped by indication, in whom days ranged from a median of 40 days for patients with CHF to 73 days for patients with NICM, a difference equivalent to roughly 1 month. In fact, when examined by reason for WCD prescription, days of wear and age were highly correlated. Comparison between median days of wear by WCD indication and age indicated a Pearson correlation of 0.89 for the 6 indications of DCM, ICM, NICM, ICD explantation, myocarditis, and CHF, which combined for 98% of the patient population.

Comparison of patients in the first quartile of daily use (≤21.0 hours) with the upper 3 quartiles revealed younger median age (53 years [interquartile range, 43–61 years] versus 59 years [interquartile range, 49–69]; *P*<0.001). The median days of wear for patients in the first quartile differed by 12 days compared with the upper 3 quartiles (50 days [interquartile range, 24–83 days] versus 62 days [interquartile range, 37–92 days]; *P*<0.001). When grouped by primary cardiac indication, DCM occurred more frequently among the lower quartile of daily use, that is, 618 (41%) versus 1585 (35%; *P*<0.001). On the other hand, ICM and ICD explantation were observed less frequently among the patients in the lower quartile, occurring in 352 (23%) versus 1252 (28%; *P*<0.001) and 145 (10%) versus 561 (13%; *P*<0.01), respectively. The remaining indications were unchanged when the lowest and upper 3 quartile groups were compared.

The relationship between total days of wear and median daily use also was evaluated. Patients wearing the WCD for a greater number of days exhibited progressively increasing hours of daily use. For example, there were 179 patients (equivalent to 3% of the patient population) with ≤3 total days of WCD wear, and these patients exhibited a median hourly daily use of 17.6 hours (interquartile range, 12.1–23.4 hours). In contrast, 189 patients (3%) had 4 to 7 days of wear, and these patients exhibited median daily use of 22.1 hours (interquartile range, 16.1–23.6 hours; *P*<0.01). Among the 5692 patients (94%) wearing the WCD for >7 days, median daily use was 23.2 hours (interquartile range, 21.3–23.7 hours), ≈97% of the hours available in a day.

### Appropriate WCD Treatment

In total, 120 patients were treated with 163 shocks ranging from 1 to 5 shocks per episode. Ninety-four patients were shocked in response to VT/VF during a cumulative wear time of 1124 patient years, corresponding to an appropriate treatment frequency of 1.6 (95% confidence interval [CI], 1.3–1.9) per 100 patients and incidence rate of 8.4 (95% CI, 6.8–10.2) per 100 years of wear (Table 3). Seventy percent of appropriate treatments occurred within the first 40 days of WCD use, and 89% of all treatments occurred within 90 days of the first day of wear. Appropriate treatments resulted from detection of VT in 62% and VF in 38% of cases. Episodes of VT included 4 instances of monomorphic VT that degenerated into VF after treatment, resulting in additional treatments from the WCD. In total 11% of episodes required >1 shock to terminate. Examination of the remaining ECG records revealed that the device detected 242 episodes of VT occurring in 70 patients during which patients responded by pressing the response buttons. These episodes were self-terminating VT, and as a result of patient interaction, shock treatment was safely withheld.

Evaluation of treated patients by sex illustrated that the appropriate shock incidence rate was 8.5% (95% CI, 6.7–10.7) for men and 7.9 (95% CI, 4.8–12.3) for women (Table 3). Evaluation of treated patients by age or by primary cardiac indication leading to WCD prescription revealed that, except for patients within the fourth quartile of age (>68 years) and ICD explantation patients, patient subgroups were statistically similar with regard to the incidence rate of appropriate treatment. For example, with the χ^2^ test, the number of treated patients in the >68-year age group versus all other patients had a value of *P*<0.05 and an odds ratio of 1.7 (95% CI, 1.1–2.6). Evaluation of treated patients among the ICD explantation subgroup versus all other patients resulted in a value of *P*<0.001 and an odds ratio of 2.5 (95% CI, 1.5–4.0).

**Table 3.**
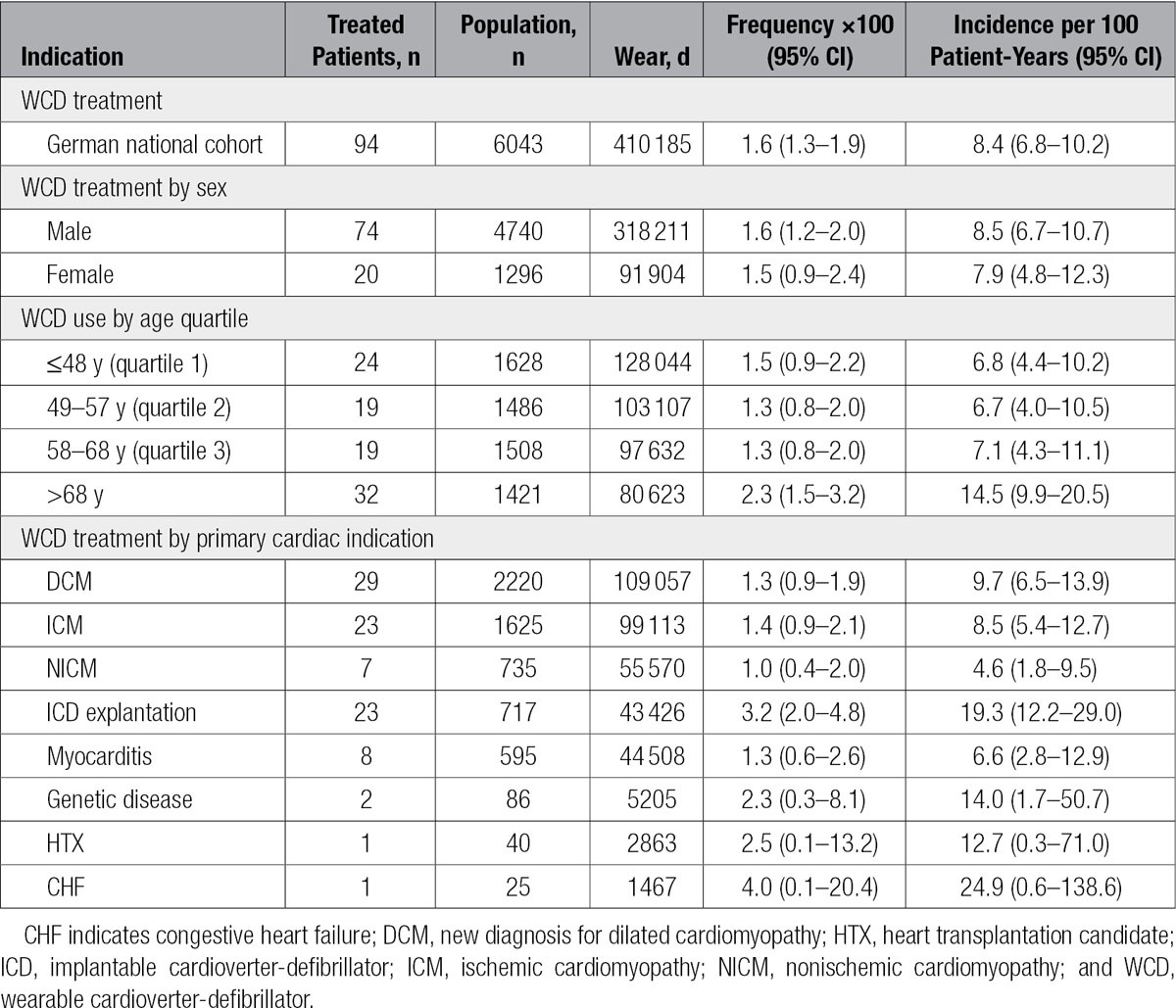
WCD Appropriate Treatment by Sex and Reason for Prescription

### Treatment Outcome

Overall, 88 patients (94%) were converted successfully into a slower heart rhythm such as normal sinus rhythm. Survival 24 hours after shock was achieved by 87 treated patients (93%). Of 6043 patients, there were 7 deaths within 24 hours after defibrillation of treatable arrhythmia, 4 as a result of unresponsive monomorphic VT and 3 caused by VF. There was 1 additional death after sustained asystole. When all patient deaths were examined by sex, there were 7 male patients and 1 female patient. Half of the deaths occurred among ICD explantation patients, with the remaining distributed among the DCM, post-MI, and recent percutaneous coronary intervention groups (Table 4).

**Table 4.**
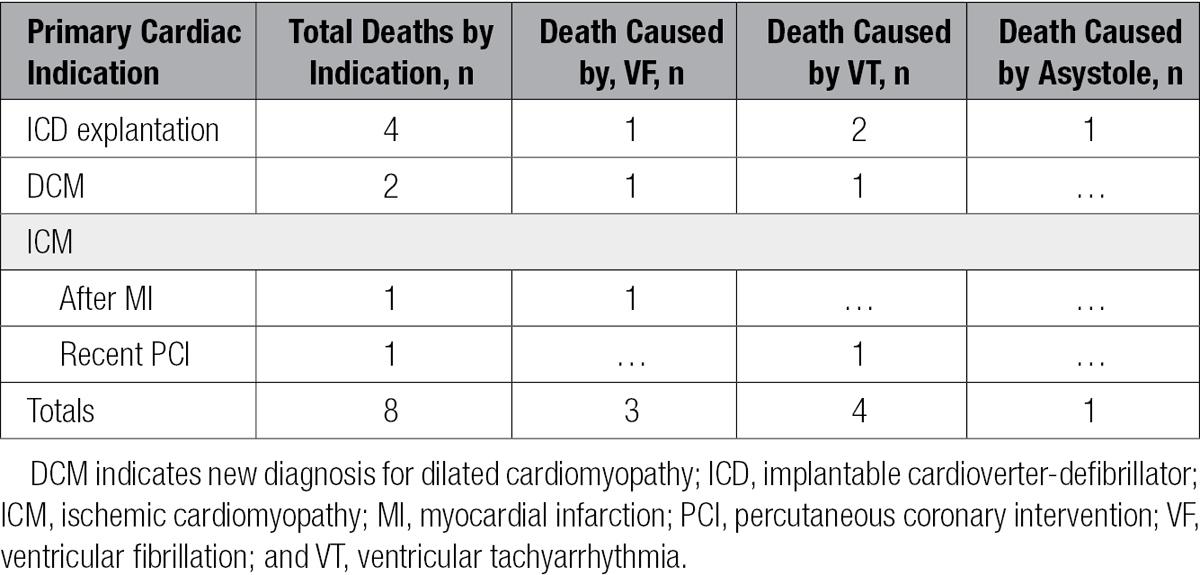
Patient Deaths by Indication

### Inappropriate Shock Treatments

Shock treatment for reasons other than treatable VT/VF arrhythmia occurred in 26 patients (0.4%), an incidence rate of 2.3 (95% CI, 1.5–3.4) per 100 patient-years, and was primarily the combined result of signal artifact, fast supraventricular tachycardia, and patient decision not to interrupt the treatment sequence by pressing the WCD response buttons. Supraventricular tachycardia was the reason for inappropriate treatment in 10 instances. Inappropriate detection by the WCD and failure to press the response buttons by the patient occurred in 13 instances of inappropriate treatments, with signal artifact, multicounting on normal ECG signal, and ECG signal loss accounting for 8 instances, 4 instances, and 1 instance, respectively. In 1 case of inappropriate treatment preceded by the device alarm, the patient did not press the response buttons. Additionally, as a consequence of asystole, 2 treatment episodes occurred, presumably resulting from the ECG baseline being interpreted as fine VF, with 1 associated patient death that was attributed to asystole by medical staff with direct knowledge of the case. No deaths were linked to the delivery of inappropriate shocks if asystole was not already present.

## Discussion

The salient findings of the present study confirm the previously published conclusion that the WCD can save lives in vulnerable patients. This study also extends the description of the usefulness of the device to a healthcare system outside the United States where patient treatment strategies vary such as the extensive use of myocarditis as a diagnosis in Germany, and the accrued 6043 patients make up the second largest study cohort to date investigating WCD use among patients at risk for sudden cardiac arrest.

Previously published studies have shown that the WCD successfully identifies and terminates VT/VF. For example, in a study involving 3569 patients, 59 patients (1.7%) were shocked in response to 80 episodes of sustained VT or VF, with first shock success reported in 76 of 76 patients (100%) with unconscious VT/VF and 79 of 80 patients (99%) with any VT or VF.^15^ The effectiveness of the WCD was reproduced in a study of 8453 patients in which 133 patients (1.6%) received shock for VT/VF and 91% were resuscitated.^16^

This analysis evaluated German experience with the WCD from 2010 to 2013. The data confirm good compliance of patients with wearing the device. An examination of daily use in the cohort indicated that the majority of patients (97%) wear the device during most activities of daily living. Patients are encouraged to remove the WCD only during bathing, and the overall hours of wear suggest that for the majority of patients this is indeed the case.

A comparison of days of wear with hours of daily use indicated that patients with more days of cumulative wear had increased hours of daily use, and this is consistent with previous evaluations of WCD patients.^15^ Of the 97% of German patients who wore the WCD for at least 4 days, the median daily use exceeded 22.1 hours, corresponding to at least 92% of the maximum hours available. Similarly, when days of wear exceeded 1 week, corresponding to 94% of patients, the median daily use reached 23.2 hours (97%), an increase of 1.1 h/d. This result is consistent with that reported for 3569 US patients in whom the median daily use was 21.7 hours.^15^ The correlation between days of wear and increasing daily use is suggestive of increased adherence to other therapies for these patients such as medication use, compliance with follow-up appointments, and lifestyle choices.

None of the previous large studies evaluated differences in total days of wear with respect to indication leading to WCD prescription. This analysis presents a difference of ≈1 month, ranging from a median of 40 days for patients with CHF and 48 days for patients with ICD explantation to 73 days for patients with NICM. These differences are arguably the result of variable diagnostic pathways and therapy strategies. For example, although WCD use among ICD explantation patients serves as a bridge to reimplantation and the observed time delay likely reflects the waiting time to reimplantation after ICD device removal owing to infection, the longer days of use recorded from the NICM patient group are likely related to longer time periods encountered before full evaluation of the patient’s chances for recovery.

In the national cohort overall, 1.6% of patients received shock treatment for VT/VF, which is comparable to the 1.7% and 1.6% reported by other investigations.^15,16^ Sudden cardiac arrest occurred among patients with different types of cardiomyopathy, suggesting that all patient groups benefit from the WCD.

Of the 94 patients who received treatment for VT/VF, 88 (94%) successfully converted into a slower heart rhythm. The rates of conversion and acute survival were similar to those reported in previous WCD patient studies.^15^ Of treated patients, 89% required 1 treatment shock by the WCD to terminate VT/VF, so shock efficacy among this cohort is similar to that reported in ICD studies (up to 80%–90%^17^) and previous WCD studies (75%–99%^1,15^). In contrast to large ICD trials,^3,5^ the examination of patients by sex revealed similar appropriate shock rates for men and women. However, 8 deaths occurred during the study. Analysis of these patients failed to indicate a correlation between death and patient subgroup characteristics.

ICD trials have reported that reductions in shock treatment correlate with reductions in all-cause mortality.^18^ The German cohort analysis uncovered 242 sustained but self-terminated episodes of VT among WCD patients^19^ that were not treated safely because conscious patients held the response buttons. Some of the self-terminated VT episodes were >8 minutes in duration without loss of consciousness by the WCD user.

The 3.6-fold difference in appropriate and inappropriate shocks exceeded that reported in previously published WCD studies. In the German cohort, 26 patients (0.4%) were shocked inappropriately, whereas in previous studies, 1.9% and 1.2% of patients were reported as having received inappropriate shocks.^15,16^

By presenting data obtained from patients recruited from 404 different cardiology centers located throughout Germany, this study suggests that the recruited patients represent the German national experience. Furthermore, this study demonstrates that the usefulness of the WCD in clinical practice extends to a variety of cardiovascular indications and that guideline recommendations for WCD use should be expanded.

### Limitations

The definitions and hierarchy used to assign patients to a single cardiac diagnosis likely resulted in overlap among disease types. Inherent to the study design of a retrospective, nonrandomized, observational analysis is the possibility of selection bias. Follow-up data on death beyond 24 hours after treatment were lacking, as were data on cardiac comorbidities and LVEF, precluding analysis of these factors. During the patient accrual period, there were no guidelines in Germany to identify patients who might best benefit from WCD prescription instead of immediate ICD implantation. In fact, the WCD has been prescribed for a wide range of patients in whom the physician determined an elevated risk of SCD. Therefore, prescribing physicians may have influenced patient selection in ways undeterminable with the data available.

### Conclusions

The German Cardiology Association and the European Society of Cardiology have recently published recommendations for use of the WCD.^20,21^ In agreement with the experience gained from the German national cohort that the WCD can save lives in vulnerable patients, WCD use was recommended for patients with poor left ventricular function who are at risk of SCD but are not candidates for ICD therapy. WCD use was given a Class IIa-C recommendation for use directly after an ICD explantation when reimplantation of an ICD is not possible, as a bridge to transplantation for patients on the waiting list for cardiac transplantation without an ICD, and for use during the acute phase of myocarditis until full recovery or ICD implantation.^21^ WCD use was given a Class IIb-C recommendation for continued use among myocarditis patients expected to achieve improved left ventricular function^20^ and for individual selection of patients early after revascularization by percutaneous coronary intervention or coronary artery bypass graft, recent acute MI patients, and patients with DCM and NICM when there is an expectation of improvement in LVEF.^20,21^ In the German national cohort, non–ICD explantation patients made up 85% of WCD use, and they experienced sudden cardiac arrest at rates equivalent to those in the United States. These sudden cardiac arrest rates were both nontrivial and comparable among the diagnostic groups. Moreover, it is unclear how to predict reliably which patients will see improvement in their LVEF. The German national experience with the WCD supports the German Cardiology Association and the European Society of Cardiology recommendations for use in patients who are at high risk of SCD.

## Sources of Funding

The work was funded from a grant by ZOLL.

## Disclosures

Drs Szymkiewicz and Ringquist are used by ZOLL. Drs Strasser and Günther received referent honoraria from ZOLL. The other authors report no conflicts.

## Clinical Perspective

### What Is New?

- The first large, nationwide study outside the United States extends wearable cardioverter-defibrillator (WCD) use to a healthcare system outside the United States in which patient treatment strategies vary such as the extensive use of myocarditis as a prescribing diagnosis in Germany.
- This is the first European analysis supporting the recently published recommendations of the German Cardiology Association and European Society of Cardiology for WCD use.

### What Are the Clinical Implications?

- The study is initiating discussion to include using the WCD for patients with temporary high risk of sudden cardiac death and normal left ventricular function such as before establishing a treatment path for genetic sudden cardiac death syndromes and during optimization of antiarrhythmic drug therapy.
- Because implantable cardioverter-defibrillator implantation is frequent in Germany, use of the WCD provides time for patients and physicians to safely seek alternatives to implantation.
- The study confirms the overall value of the WCD in the detection and termination of ventricular tachyarrhythmia/ventricular fibrillation among outpatients independently of patient or bystander intervention.
